# Perspectives and Challenges in the Fight Against COVID-19: The Role of Genetic Variability

**DOI:** 10.3389/fcimb.2021.598875

**Published:** 2021-03-15

**Authors:** Mariana Guilger-Casagrande, Cecilia T. de Barros, Vitória A. N. Antunes, Daniele R. de Araujo, Renata Lima

**Affiliations:** ^1^ Institute of Science and Technology, São Paulo State University–UNESP, Sorocaba, Brazil; ^2^ Laboratory for Evaluation of the Bioactivity and Toxicology of Nanomaterials, University of Sorocaba-UNISO, Sorocaba, Brazil; ^3^ Human and Natural Sciences Center, Federal University of ABC, Santo André, Brazil

**Keywords:** polymorphism, immune response, ACE2 receptor, cytokines, chemokines, X chromosome, SARS-CoV-2, coronaviruses

## Abstract

In the last year, the advent of the COVID-19 pandemic brought a new consideration for the multidisciplinary sciences. The unknown mechanisms of infection used by SARS-CoV-2 and the absence of effective antiviral pharmacological therapy, diagnosis methods, and vaccines evoked scientific efforts on the COVID-19 outcome. In general, COVID-19 clinical features are a result of local and systemic inflammatory processes that are enhanced by some preexistent comorbidities, such as diabetes, obesity, cardiovascular, and pulmonary diseases, and biological factors, like gender and age. However, the discrepancies in COVID-19 clinical signs observed among those patients lead to investigations about the critical factors that deeply influence disease severity and death. Herein, we present the viral infection mechanisms and its consequences after blocking the angiotensin-converting enzyme 2 (ACE2) axis in different tissues and the progression of inflammatory and immunological reactions, especially the influence of genetic features on those differential clinical responses. Furthermore, we discuss the role of genotype as an essential indicator of COVID-19 susceptibility, considering the expression profiles, polymorphisms, gene identification, and epigenetic modifications of viral entry factors and their recognition, as well as the infection effects on cell signaling molecule expression, which amplifies disease severity.

## Introduction

**Figure 1:**
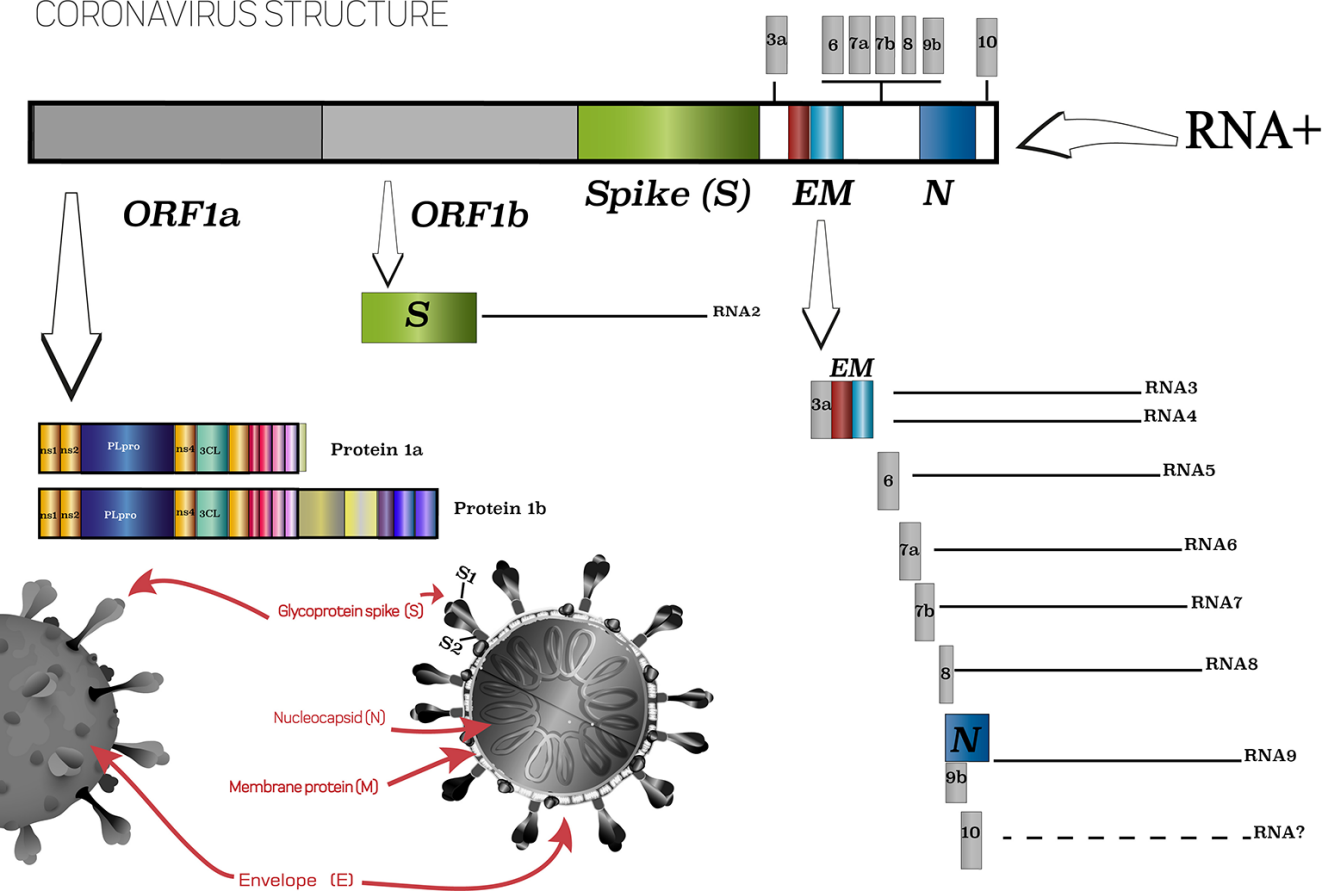
Scheme of SARS-CoV-2 virus and its main characteristics. Based on ViralZone, 2020.

COVID-19, the disease caused by the new coronavirus SARS-CoV-2, was first reported on December 29, 2019 in the city of Wuhan, China (WHO, 2020), and since then, more than a year later, we are living through a pandemic that has challenged doctors and scientists worldwide. SARS-CoV-2 has already infected 106,797,721 people and caused more than 2,341,145 deaths worldwide (WHO, 2021), with the main problem being the immune response that occurs in individuals. This is due to some peculiarities that SARS-Cov-2 presents, in particular the use of the angiotensin-converting enzyme 2 (ACE2) receptor to insert into human cells. Molecular study of the virus showed that it is a single-stranded RNA virus with a positive envelope (29.8 kb) that has six common open reading frames (ORFs) and several accessory genes (Mahmoudabadi and Phillips, 2018; Zheng, 2020) (**Figure 1**).

Sequence analysis showed that the viral genome found in different patients was highly conserved (Lu et al., 2020a; Zhou et al., 2020), indicating a recent evolution. The amino acid sequence of the experimental receptor binding domain of SARS-CoV-2 resembles that of SARS-CoV, the virus that triggered an outbreak in 2003, indicating that these viruses recognize the same receptor (Ren et al., 2020). Knowing the origin and structure of the new coronavirus, as well as its interaction with human cells, was a very important step for the confrontation that is currently being waged.

It is well known that while there are cases of individuals who are resistant to SARS-CoV-2 infection or asymptomatic, there is also a high rate of patients who progress to the severe form of the disease and die. This reality triggers several provocative questions.

## What Leads SARS-CoV-2 to Cause People Major Problems?

ACE2 enzyme acts as a receptor responsible for viral binding to the cells (Wrapp et al., 2020; Xu et al., 2020). A peculiarity of SARS-CoV-2 is the surface protein (Spike, S) (**Figure 1**), which mediates virus recognition by human cells, that has a 10- to 20-fold higher affinity to ACE2 in comparison to the surface proteins of SARS-CoV, which contributes to its high infection and dissemination rates (Gheblawi et al., 2020). Such differences occur due to structural changes resulting from a four-residue motif (residues 482–485: Gly-Val-Glu-Gly) in the binding region, which makes the protein ridge more compact and allows better contact with the hACE2 N-terminal helix (Yan et al., 2020; Yi et al., 2020). Those structural features are essential to understand how ACE2-SARS-CoV-2 binding is processed.

In this scenario, the comprehension of ACE2 physiological functions and specific features could explain how comorbidities (hypertension, diabetes, obesity, and immunological diseases) and other biological factors (older age, male gender) can contribute to enhance the symptoms’ severity, with progression to death, evoked by COVID-19.

The ACE2 receptor exerts a pivotal role by regulating the degradation of Ang II in Ang 1-7 *via* the G-protein accoupled Mas receptors axis, especially in the lungs. The protective functions associated with the ACE2 axis is a result of Ang 1-7 production, which leads to anti-inflammatory effects, the reduction of lymphocytes and neutrophils infiltration with decreased perivascular and bronchial inflammation. These effects are counter-regulated by AngII-mediated responses, such as myocardial and endothelial disfunction, hypertension associated with obesity, and coagulation alterations (Verdecchia et al., 2020).

**Figure 2:**
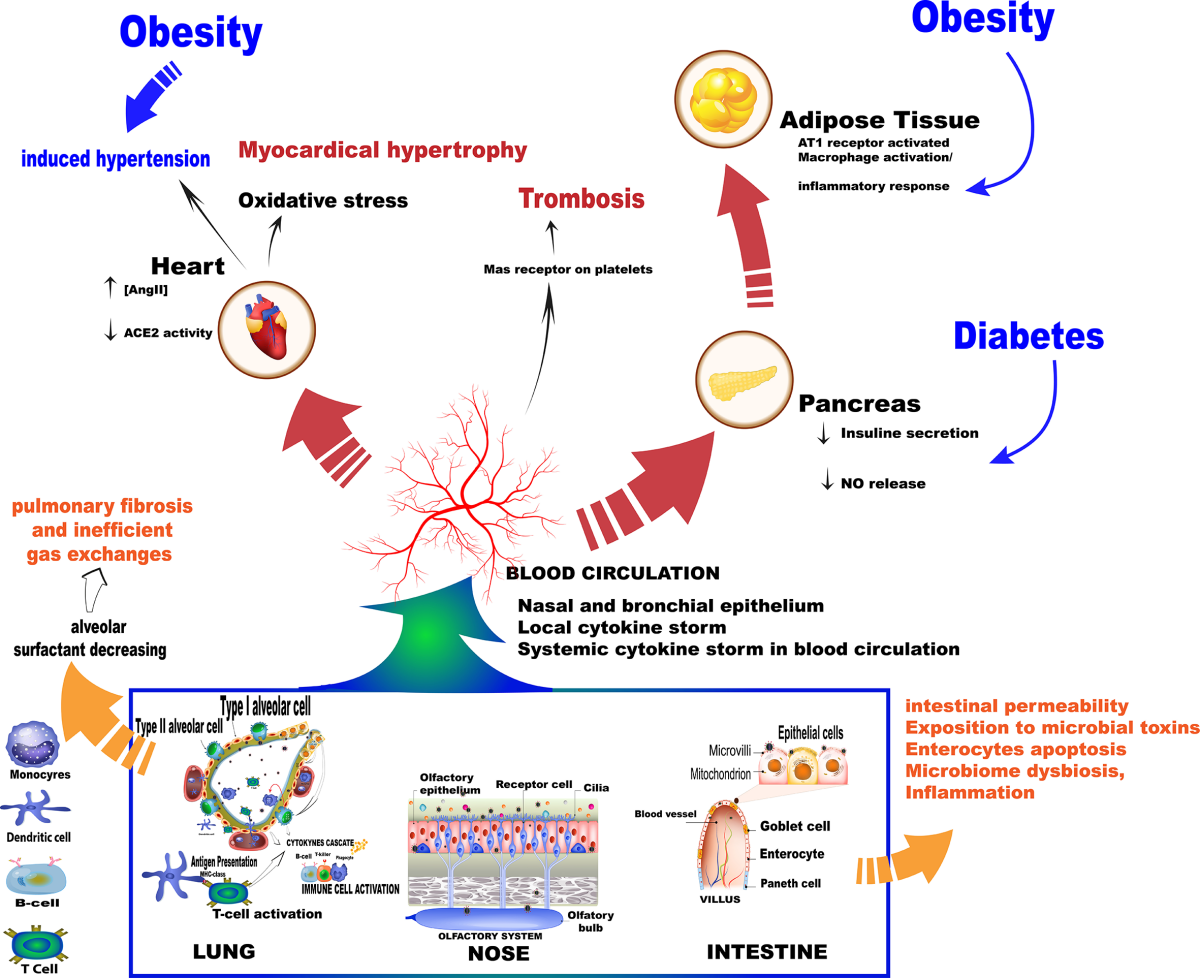
Summary of the main events as a result of the SARS-Cov-2 virus entry into the body. Once in contact with the cells the virus triggers mechanisms that increase the production of cytokines, which in turn trigger an immune cascade.

Conversely, impaired ACE2 function in the lungs is responsible for increasing free-bradykinin levels, activating pro-inflammatory cytokines release, and pulmonary injury. Notably, Mas receptor activation stimulates prostacyclin and nitric oxide (NO) release in platelets, evoking anti-thrombotic effects. Additionally, Mas receptors are also expressed in the pancreas, improving perivascular blood flow, and in cardiac adipocytes, preserving cardiac function (Hoffmann et al., 2020; Liu et al., 2020). In this sense, it was possible to predict that the deficiency of or a reduction in the physiological function of ACE2 may favor pulmonary inflammation, thrombosis, obesity-induced hypertension, adipose tissue inflammation, and cardiac failure, which is especially detrimental to the baseline risk of COVID-19 patients (**Figure 2**).

Different morbidities, such as obesity, old age, and chronic obstructive pulmonary disease (COPD) are associated with severe COVID-19 symptoms, which, in general, are supra regulated by dipeptidyl peptidase-4 (DPP-4) (Roy et al., 2020). In addition, both DPP-4 and ACE2 are dysregulated proteins in diabetes, so it may be possible for diabetic patients to present more severe cases of COVID-19 due to the increased levels of these proteases (Valencia et al., 2020). Studies have also shown that ACE2 overexpression occurs in the nasal and oropharyngeal epithelium of individuals who are more vulnerable to severe COVID-19 symptoms (Chua et al., 2020; Li et al., 2020a; Ziegler et al., 2020).

Hamming et al. (2004) investigated the presence of ACE2 in different tissues and observed that this receptor is highly abundant in the lung and small intestine epithelium, as well as in the vascular endothelium. Those observations were reinforced by findings on the overexpression of ACE2 in alveolar epithelial cells and enterocytes, which compose potential target organs of SARS-CoV-2 (Zhou et al., 2020). Specifically, expression of the ACE2 gene is concentrated in type I (oxygen and carbon dioxide exchanges) and II pneumocytes (alveolar surfactant production), and the profile of gene expression varies from individual to individual (Zhao et al., 2020). In the presence of SARS-CoV-2 infection, pneumocyte types I and II are damaged, decreasing the production of alveolar surfactants, which implies reduced pulmonary elasticity and pneumocytes type I repair. These factors result in pulmonary fibrosis and inefficient gas exchange (Verdecchia et al., 2020).

The large contact surface of alveolar epithelial tissue associated with the high expression levels of SARS-CoV-2 entry factors in bronchial secretory cells and nasal epithelial cells emphasizes that some individuals are more vulnerable to respiratory failure than others. Additionally, microarray database analysis reported that infection-related factors are also highly expressed in the gastrointestinal system, explaining the diarrhea and virus isolation from stool samples (Gkogkou et al., 2020). Therefore, the respiratory and gastrointestinal tracts represent the most frequent routes of virus access to the body. A structural study showed that, in addition to showing high levels of expression in the small intestine, ACE2 also has significant expression in the testicles, kidneys, heart, thyroid, and adipose tissue, with lower expression in the spleen, bone marrow, brain, blood cells, blood vessels, and muscles (Li et al., 2020a).

Initially, SARS-CoV-2 mediated infection is dependent on the viral S1 domain binding to ACE2 and their cleavage by the transmembrane serine protease 2 (TMPRSS2), whereas the viral S2 domain is responsible for conformational changes that drive the fusion process between viral and host cell membranes (Brest et al., 2020; Wang et al., 2020a). Those events are followed by virus entry and its replication in the cells (Devaux et al., 2020; Wang et al., 2020a). However, other molecular factors have been implicated as essential not only for viral entry but mainly for infection progressive cellular effects, such as the membrane anchored metalloproteinase domain-containing protein 17 (ADAM 17), which evokes the ACE2 shedding mechanism (Brest et al., 2020; Gheblawi et al., 2020).

Virus binding induces ACE2 deficiency, amplifying the dysregulation between the ACE-Ang II-AT1 (type 1 receptor for angiotensin II) receptor and the ACE2-Ang 1-7-Mas receptor axis since there is a reduction in ACE2 catalytic properties favoring the ACE-Ang II-AT1 receptor pathway. Moreover, the renin–angiotensin–aldosterone system imbalance evokes pronounced detrimental effects in all COVID-19 stages, since multiple organ failure and a hyperinflammatory response are the main clinical findings in severe cases (Mahmudpour et al., 2020).

**Figure 3:**
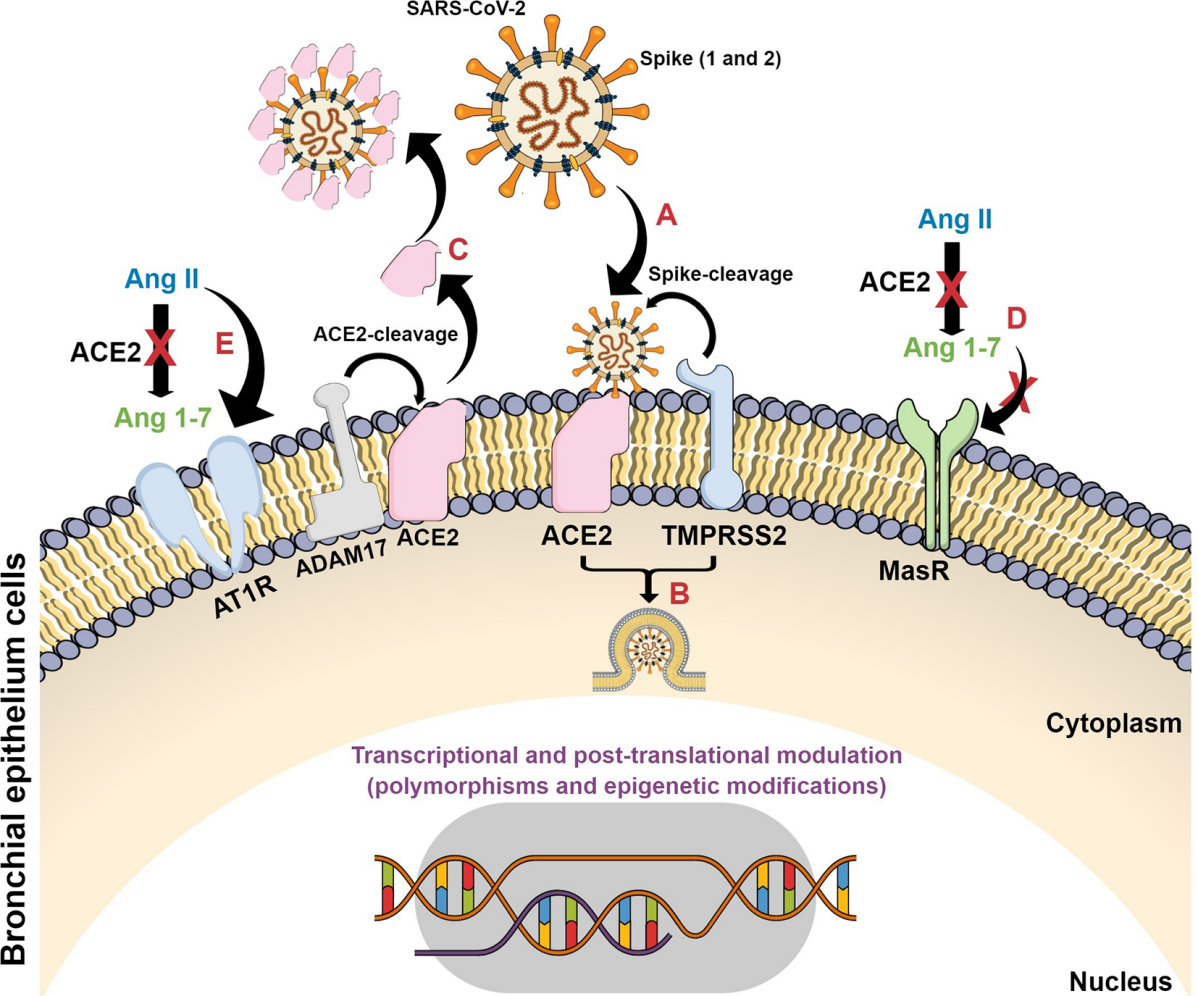
Schematic representation of SARS-CoV-2 infection in target cells and its consequences on ACE2 signaling pathways. **(A)** SARS-CoV-2 spike proteins bind to ACE2 with their consequent cleavage by ACE2 and TMPRSS2; **(B)** viral fusion, internalization and release of viral genetic material; **(C)** ACE2 cleavage by ADAM17 forming a plasm-soluble ACE2 isoform and its binding on viral particle at non-target tissues; **(D)** The conversion of Ang II to Ang 1-7 is blocked with subsequent decrease on Mas receptor protective axis; **(E)** Ang II interacts with AT1 receptor and triggers detrimental pathways evoking inflammatory processes.

The dysregulation of the ACE2/Ang II/AT1R axis, attenuation of the ACE2/Mas receptor axis, and increase in the activation of the ACE2/bradykinin B1R/DABK pathway and their complementary cascades are the main molecular interactions that support the cytokine storm (Hirano and Murakami, 2020; Mahmudpour et al., 2020). Once the virus binds to ACE2, this enzyme does not convert Ang II into Ang 1-7, making it impossible to activate MasR and form the complex to control inflammation. In the absence of Ang 1-7, there is also an increase in the expression of p38 MAPK and NFk-β pathways and inflammatory factors, as well as the binding of Ang II to the AT1R receptor, triggering pro-inflammatory responses and, as a result, pulmonary deterioration (Gheblawi et al., 2020; Mahmudpour et al., 2020; South et al., 2020) (**Figure 3**).

The increase in Ang II and the consequent activation of its AT1R receptor also hyperactivate the complement system cascade, causing even more inflammatory responses, inducing vasoconstriction, increasing oxidative stress, and promoting inflammation and fibrosis (Risitano et al., 2020; South et al., 2020). In addition, there is an increase in the activity of the inflammatory lung factor [des-Arg9]-BK (DABK), which also causes the exacerbated release of cytokines (Tolouian et al., 2020). These events justify the relationship between a high viral load, severe lung damage, and Ang II levels in serum samples from patients infected with SARS-CoV-2 (Liu et al., 2020).

Lymphopenia is one of the immunological responses characteristic of severe cases of COVID-19 accompanied by a marked decrease in the absolute number of circulating CD4+ cells, CD8+ cells, B cells, and natural killer cells (NK) (Zhou et al., 2010; Tan et al., 2020a), as well as a decrease in other cell types and increased levels of pro-inflammatory cytokines (Moore and June, 2020; Verdecchia et al., 2020). The binding of SARS-CoV-2 with the ACE-2 receptor and the consequent change in the ACE-cascade triggers several molecular processes that lead to the overproduction of cytokines and hyperinflammation, and the activation of some cells that play a role in the immune system (Dhochak et al., 2020; Mahmudpour et al., 2020).

## An Explosive Response

The dysregulation of the immune system triggers the evolution of COVID-19 to its severe form in some patients (Giamarellos-Bourboulis et al., 2020; Huang et al., 2020), and respiratory failure is generally associated with this characteristic and not necessarily with increased viral load (Risitano et al., 2020). The simultaneous activation of both innate and adaptative immune systems through the binding of SARS-CoV-2 to the cell receptor makes the immune system reaction more damaging to the cells than the virus itself. In this way, the events following infection lead to severe inflammatory disorders with a possible progression to death (Du et al., 2020; Zhang et al., 2020a).

A study by Tan et al. (2020b) concluded that a stronger antibody response is associated with delayed viral clearance and consequently more severe symptoms of the disease. Patients with COVID-19 and lower IgG titers experienced faster viral clearance when compared to patients with COVID-19 and higher IgG titers. Another study showed that asymptomatic individuals had lower IgG titers compared to those that were symptomatic (p=0.005) 3–4 weeks after exposure to SARS-CoV-2, and this difference remained during the convalescent phase (p=0.02), 8 weeks after hospital discharge (Long et al., 2020). This imbalance in immune response may occur due to a mechanism known as antibody-dependent enhancement (ADE), where there is an increase in pathogenicity in the presence of sub-neutralizing and non-neutralizing antibodies that facilitate viral entry into cells, triggering an increase in infection and virulence (Bournazos et al., 2020). Other immunological studies on SARS-CoV suggest that anti-SARS-CoV-2 antibodies may intensify COVID-19 symptoms through the ADE mechanism, increasing infection or triggering detrimental immunopathology (Lee et al., 2020).

According to Arvin et al. (2020), this phenomenon occurs when non-neutralizing or sub-neutralizing antibodies bind to viral antigens without blocking or eliminating infection and, in an unexpected way, facilitate viral entry into the cells through the interaction with crystallizable fragments or receptors’ complements. Even if there is no active viral replication, the facilitated viral entry into cells can lead to the activation of macrophages, monocytes, and B cells and the production of tumor necrosis factor alpha (TNFα) and interleukins 6 and 10 (IL-6 and IL-10), affecting disease prognosis, an approach previously observed by Taylor et al. (2015). This event may be responsible for the increase in the number of immune cells observed in patients with a poor prognosis.

The ADE mechanism is mediated by immunoglobulin receptor Fc(R), which has a pivotal role in immune response, controlling innate and humoral immunities that are essential for responses against infections and the prevention of chronic inflammation and autoimmune diseases. In addition, these immunoregulatory processes may play a role in the pathogenesis of the disease, triggering responses such as the release of cytokines and phagocytosis. Notably, these receptors have a large genetic polymorphism, with a variation in the immune response according to the presented gene (Hargreaves et al., 2015; Bournazos and Ravetch, 2017), which may contribute to a larger or smaller immune response regarding infection by SARS-CoV-2.

A study by Sun et al. (2020) reported that changes in peripheral blood inflammatory cells may signal the severity and spread of COVID-19. In this study, patients with severe infection showed a decrease in the number of lymphocytes, eosinophils, basophils, and an increase in neutrophil count. A decrease was observed in the total count of T lymphocytes (T CD4+, T CD8+), as well as NK cells 2 weeks after the treatment of patients who posteriorly died. Although there is a decrease in CD4 and CD8 lymphocyte cells and NK cells in the most severe cases of COVID-19, hyperactive circulating monocytes continue to produce proinflammatory cytokines, such as TNFα and IL-6. The hyperactivation of monocytes, decrease of lymphocytes, and high IL-6 release causes low expression of the cell surface receptor encoded by the human leukocyte complex (HLA-DR). As a result, there may be a failure in antigen presentation preventing the action of the immune system against the virus, followed by hyperinflammation due to the high production of pro-inflammatory cytokines (Giamarellos-Bourboulis et al., 2020).

In the study performed by Qin et al. (2020), most patients with severe COVID-19 symptoms showed an increase in cytokine and chemokine serum levels, in addition to high production of neutrophils and low production of lymphocytes, indicating that the hyperinflammatory response was responsible for the severity of the disease. The impaired immune system, with damaged T lymphocytes, may cause greater susceptibility to secondary pulmonary bacterial infections, which justifies the use of some antibiotics as an adjunctive pharmacological therapy.

Thus, a cytokine storm is one of the main characteristics of severe cases of SARS-CoV-2 infection, being associated with the severity of the disease and mortality (Giamarellos-Bourboulis et al., 2020; Lu et al., 2020b). It involves the uncontrolled release of a large amount of pro-inflammatory cytokines, such as TNFα, IL-6, and IL-1β, in a systemic way (Roshanravan et al., 2020). This reaction is formed by complex interconnected networks that interact in different ways, showing us that there are several molecular mechanisms to be unveiled (Mahmudpour et al., 2020). In summary, the consequence of infection by SARS-CoV-2 is that patients have increased serum levels of IL-6, IL-1β, IL-2, IL-8, IL-17, colony stimulating factor of granulocytes (G-CSF), granulocyte and macrophage colony stimulating factor (GMCSF), interferon gamma-induced protein 10 (IP-10), monocyte-1 protein-chemoattractant-1 (MCP-1), chemokine 3 ligand (CCL3), and TNFα (Scheller and Rose-John, 2006; Mehta et al., 2020).

In this way, the reason why SARS-CoV-2 differentially changes the organism’s response and which strategies organisms use to fight infection are questions to be elucidated. According to Ulhaq and Soraya (2020), there is evidence that the circulating IL-6 levels are intricately linked to the severity of infection in patients with COVID-19, especially in those with lung injury. IL-6 is a pleiotropic multifunctional cytokine that plays an important role in human metabolism (Hunter and Jones, 2015). Its action is mainly linked to lymphocyte proliferation, antibody production and multiplication, and differentiation of hematopoietic cells, targeting B cells, T cells, neutrophils, eosinophils, and basophils. IL-6 is associated with diseases including leukemia, hypertension, atherosclerosis, and different types of malignant tumors. The greatest production of this cytokine occurs in T cells, endothelial cells, fibroblasts, macrophages, and monocytes (Brennan and Foey, 2002). An increase of pro-inflammatory cytokines was detected in the serum of patients infected by coronaviruses SARS-CoV and MERS-CoV, being related to inflammation and lung damage (Wong et al., 2004; Mahallawi et al., 2018).

## Understanding the Course of the Immune Response

As previously discussed, in addition to respiratory failure due to pulmonary inflammation, patients with severe COVID-19 symptoms may have systemic inflammation that causes damage to multiple organs, with distinct immune reactions among individuals. A current study, named ENE-COVID, which is being performed by the Spanish Ministry of Health and the Carlos III Institute of Public Health, showed that the majority of Spain’s population, one of the European populations most affected by COVID-19, had negative serology for SARS-CoV-2. Only 5% of more than 61,000 participants in the study had serum prevalence for IgG antibodies, of which one third were asymptomatic. Among the patients with positive results for COVID-19 as determined by qRT-PCR, 90% of serum prevalence was observed 14 days after diagnosis. From these results, it was concluded that herd immunity cannot be achieved without major side effects and a high mortality rate in the susceptible population (Pollán et al., 2020).

In a study by Seow et al. (2020), serum samples were collected from 65 patients diagnosed with COVID-19 by qRT-PCR and from 31 healthcare workers with positive serology and analyzed sequentially up to 94 days after the appearance of symptoms for neutralizing antibody responses. Eight days after the onset of symptoms, 95% of patients diagnosed with COVID-19 showed serum conversion and neutralizing antibody activity, with greater antibody responses in the most severe cases of the disease. However, over time, more than 60 days after the onset of symptoms, the antibody levels of most studied participants decreased, with a faster decline in those who had shown low antibody activity at the beginning. This fact was also investigated by Muecksch et al. (2020), who reported that the sensibility of a given antigen-based test for N protein decreased from >95% to 85%, 61–80 days after diagnosis, and to 71%, 81–100 days after diagnosis, confirming that it is inadequate for serum prevalence studies or for individuals who show long-term chronic symptoms. Other immune mechanisms, such as long-term memory T-cell responses, play an important role in protecting against serious infections or diseases (Grifoni et al., 2020; Le Bert et al., 2020), however, the detailed analysis of responses of T cells is currently not viable in a high-performance clinical environment (Muecksch et al., 2020).

Knowing the mechanisms and long-term kinetics of antibody titers, as well as the effectiveness of serological assays, is essential for a precise interpretation of disease progression (Andersson et al., 2020; GeurtsvanKessel et al., 2020; Rosado et al., 2020); however, specific IgM and IgG antibodies are mostly investigated for the peak surface glycoprotein (S) and nucleocapsid protein (N) (Carter et al., 2020). In the future, it may be difficult to both verify seropositive individuals and study the immunization time of individuals and populations (herd immunization). In this way, new studies are developed in an attempt to improve the evaluation of the immune response through the identification of more specific regions for both early and late responses, such as ORF8 and ORF3b regions (Hachim et al., 2020; Wang et al., 2020b).

Regarding herd immunity, Britton et al. (2020) developed a mathematical model in which they evaluated how far preventive measures can lead to herd immunity for SARS-CoV-2. They applied the model in four different hypothetical populations, considering variations in age and activity levels, with the implementation of preventive measures. It was observed that the population heterogeneity, mainly related to variations in social activity, reduced the acquisition of herd immunity from 60% to 43%, considering the classic level for homogeneous populations. In the current scenario, considering the wide heterogeneity among populations, it is possible to predict the unattainability of herd immunity.

## Predicting the Future: the Genotype as an Indicator of Susceptibility

Several factors cause variations in individuals’ responses to SARS-CoV-2, including viral load, genetic susceptibility, and concomitant pathological conditions (Le Bert et al., 2020). The fact that some individuals have exacerbated inflammatory reactions and others do not gives rise to different phenotypes of the disease that are linked to genetic factors (Tisoncik et al., 2012). According to Radzikowska et al. (2020), there are different receptors that are potentially involved with SARS-CoV-2 infection in epithelial barriers and immune cells, and their differential expression, depending on age, gender, presence of other characteristics, such as obesity, smoking, and polymorphisms, and the state of the disease, can contribute to the patterns of morbidity and severity of COVID-19 symptoms.

Therefore, we must remember that individuals are genetically distinct, which can contribute to the identification of different levels of disease severity. In addition to the comprehension of the molecular mechanisms involved in SARS-CoV-2 infection, it is necessary to understand the host organism’s reaction to infection.

This raised the question: are individual differences responsible for the lethality of the disease? An initial investigation carried out by some researchers in relation to gender and age showed that there is no difference between males and females or between people of different ages in terms of infection and transmission (Scully et al., 2020; Stokes et al., 2020), which makes us believe that the individual responses after infection have great importance in determining the development of the disease (Jin et al., 2020; Mauvais-Jarvis et al., 2020; Stokes et al., 2020).

This has led researchers to several questions. Is it possible to predict a genetic composition that is less able to fight infection? Are we in a genomic era where genetic screening can identify the most resistant and the most susceptible individuals to new diseases? Could these results contribute to a more specific clinical assessment of infected individuals? To discuss these issues, the study of virus entry factors, expression profiles, polymorphisms, and epigenetic modifications may be important tools to detect susceptible individuals, which could facilitate control of COVID-19 outcomes (Anastassopoulou et al., 2020).

Since studies do not show difference between individuals in terms of transmission and infection by the SARS-CoV-2 virus, it is essential to assess individual responses and how they can be differentiated.

Almost 1 year since the start of COVID-19 pandemic, reports have shown that most patients who needed hospitalization, intensive care, intubation, and eventually died were male, with numbers 1.5- to 2-fold higher when compared to females (Mauvais-Jarvis et al., 2020). Another study showed that in relation to the studied COVID-19 cases, men showed greater complications with more severe symptoms than women (p=0.035). In the public data set, the number of men who died of COVID-19 was 2.4-fold greater than that of women (70.3% *vs*. 29.7%, p=0.016) (Jin et al., 2020).

In the United States, where the test to diagnose SARS-CoV-2 infection was prioritized for people with symptoms, the diagnostic rates were similar for both genders, but the male mortality rate was 1.5-fold higher than that of females (Stokes et al., 2020). There are reports that females have more effective innate and adaptative immunity responses to antigenic challenges (Klein, 2012; Klein and Flanagan, 2016). In addition, women respond better to different types of vaccines (Fink and Klein, 2015; vom Steeg and Klein, 2016; Fink et al., 2018).

According to Li et al. (2020b), the relationship between deaths from COVID-19 and the gender of patients may provide clues in the search for solutions to control the disease. Differences in chromosomes, genes, and hormones are influenced according to the individual’s gender which causes varied responses to several diseases, including COVID-19. Gender may be considered when analyzing clinical trials in relation to SARS-CoV-2 infection, as gender differences may reveal different approaches that are necessary for the treatment of patients with COVID-19, such as estrogen-related compounds and androgen receptor antagonists (Li et al., 2020b).

Thus, the possible polymorphisms or loss of heterozygosity of some genes in males can interfere in the response to the virus, since the fact that females have two X chromosomes could be considered an advantage. Even with the inactivation of one X chromosome, this advantage is maintained since the process occurs randomly, allowing heterozygosity, a fact that is impossible in males due to the presence of a single X chromosome. The X chromosome has the ACE-2 gene, as well as genes related to the immune system responsible for innate and adaptive immune responses (Gemmati et al., 2020). The androgen receptor (AR), toll-like receptor 7 (TLR7), toll-like receptor 8 (TLR8), kinase 1 associated with the interleukin-1 receptor (IRAK1), beta chain of cytochrome B-245 (CYBB), fork head box P3 (FOXP3), and CD40 ligand (CD40L) are among X chromosome genes involved in innate and adaptive immunity (Schurz et al., 2019).

The AR gene is responsible for the synthesis of a signal transduction protein and transcription factor, necessary for the development and expression of male phenotypes. In addition, signaling of this gene leads to several chemical activations and deactivations in the immune system, such as the inhibition of antibody production, cell differentiation *via* granulocytes, regulation of the chemotactic capacity of macrophages, regulation of T and B cell function, activation of the immune response of T cells by Th1, and inactivation of type I interferon (IFN type I) signaling pathways (Olsen and Kovacs, 2001; Lai et al., 2012; Kissick et al., 2014).

The main hormones that bind to AR are dihydrotestosterone (DHT), testosterone, androstenedione, and dehydroepiandrosterone (DHEA), which are synthesized from cholesterol. Of these four androgens, only DHT cannot be converted to estrogen, so studies using DHT are more easily interpreted. It is interesting to remember that testosterone is present in greater quantity in adult males, which can lead to a response stimulation *via* Th1. Remarkably, the AR gene has polymorphs that influence the intensity of androgen signaling, and it affects both men and women (Kissick et al., 2014). Many differences in immune cells result from exposure to androgens, showing that there is a relationship between immunity and the gender of individuals. The suppression of immune reactivity and androgen-mediated inflammation raises the threshold for the development of autoimmunity; however, further studies are still needed to elucidate these mechanisms (Bupp and Jorgensen, 2018).

The TLR7 and TLR8 genes, together with TLR4, which is on chromosome 4, mediate part of the innate immune response because they are in one of the three classes of pattern recognition receptors, the toll-like receptors (TLRs). They specifically regulate the production of type 1 IFN and other cytokines so that sexual dimorphism may be observed during antiviral responses (Meier et al., 2009). Studies carried out using female lymphocytes reported that T and B cells showed biallelic expression of TLR7 and increased transcription of immune related genes linked to the X chromosome (Souyris et al., 2018). It would explain the 10-fold higher expression of TLR in females when compared to male cells (Klein and Pekosz, 2014), as well as the lower production of INF- α. Male peripheral blood mononuclear cells produced lower levels of IFN-α after stimulation by the ligand TLR7 and higher levels of the immunosuppressive cytokine IL-10 after stimulation by ligands TLR8 and TLR9 (Torcia et al., 2012).

The IRAK1 gene is the key encoder of the intracellular signaling pathway of TLR/IL-1R and assists the recognition of viruses by TLR7/8 and the production of IFN- α, being one of the main genes that contribute to the divergence of responses related to gender after trauma and sepsis (Gottipati et al., 2008; Sperry et al., 2014). The IRAK1 haplotype contributes to differentiated immunomodulation between the genders in all ethnic groups in relation to inflammatory responses. It probably occurs due to polymorphisms in the gene (Spolarics et al., 2017).

The CYBB gene was identified as possibly responsible for the innate immune response, contributing to the activation of an intracellular oxidative explosion for the production of superoxide in phagocytes, encoding catalytic subunits of NADPH oxidase and, consequently, causing the death of microorganisms by phagocytosis (Spolarics et al., 2017; Jaillon et al., 2019).

The FOXP3 and CD40L genes cooperate in the adaptive immune response (Jaillon et al., 2019). The protein synthesized by the FOXP3 gene is a key regulator of T cell activation, more specifically Treg (regulatory T cells) that prevent autoimmunity (Mercer and Unutmaz, 2009). The protein synthesized by CD40L is responsible for transmembrane signaling involved in the activation of platelet, endothelial, and immunological cells. It acts in antigen presentation, in the activation of B cells, and in the differentiation of T cells (Elgueta et al., 2009; Spolarics et al., 2017), which could be involved in the differential development of disseminated intravascular coagulation in some patients.

In addition to those genes, several genetic factors, such as inactivation of the X chromosome and cell mosaicism, may be responsible for the hyperresponsiveness of the female immune system (Pessach and Notarangelo, 2009; Libert et al., 2010; Rainville et al., 2018). An interesting factor regarding the different mediation of the immune response in men and women is that the immune response mediated by antibodies in women is stimulated predominantly by type 2 T helper cells (Th2), whereas in men, type 1 T helper cells (Th1) are predominant (Billi et al., 2019).

The gender-bias in response to immunological problems has already been shown by Lambert (2019), considering rheumatic diseases, where it was shown that, in addition to the heterozygosity that occurs in females, different genders go through different situations since birth, with molding of the epigenome and microchimerism. The author showed that men and women are genetically, epigenetically, hormonally, and chemically different, which has an influence on the health and success of long-term treatment. A better understanding and exploration of these differences is necessary to progress in the treatment and management of all patients. There is evidence that targeting candidate genes or reversing epigenetic dysregulation according to gender may be more effective and beneficial than current therapies (Lambert, 2019). Broadening the discussion of the topic, genetic, and epigenetic differences can be parameters to assess, in response to the presence of the virus, as they can be crucial in viral recognition and individual immune response, influencing the severity of SARS-CoV-2 infection. An example of epigenetic interference is the low susceptibility of children to SARS-CoV-2 infection, which can be explained, among several factors, by the high expression of ACE-2, which in spite of being the viral receptor, ends up having a protective effect on the lungs. The high expression of ACE2 enables the binding of the receptor to Ang II (**Figure 3**) even in the presence of the virus, without altering the signaling cascade activated after the coupling of viral particles. In addition, children have a more effective innate immune system, with the ability to fight infection immediately upon infection, showing high regeneration of the alveolar epithelium (Dhochak et al., 2020). Additionally, it was found that children’s nasal epithelium, the first region of SARS-CoV-2 contact with the organism, presents low expression of ACE2, hampering virus binding (Bunyavanich et al., 2020).

In this context, other essential factors to be considered are the relationships between the levels of expression, polymorphisms, and epigenetic alterations, specifically for the viral entry factors ACE2, ADAM17, and TMPRSS2, since they are implicated in both susceptibility to viral infection in target cells and COVID-19 progressive symptoms (Hoffmann et al., 2020; Ortiz-Fernández and Sawalha, 2020).

For small bowel infections, the ACE2-TMPRSS2 and four serine protease axes also exert an essential role, being responsible for virus attachment and its internalization, explaining the gastrointestinal viral tropism (Wong et al., 2020; Zhang et al., 2020b). However, the presence of other elements on enterocytes, such as furin and B0AT1, an aminoacid transporter, can influence viral infection and its consequences. Intestinal viral infection includes the initial Spike cleavage by TMPRSS2 and furin, in segments 1 and 2, followed by S1 coupling with ACE2, while S2 mediates the membrane fusion. One of the main steps during SARS-CoV-2 bowel infection is the relationship between ACE2 and B0AT1, since B0AT1 function is regulated by the ACE2-like protein, viral coupling to ACE2 also blocks amino acid transporter activity. These effects lead to pathological conditions, similar to colitis, due to amino acid transport depletion, especially tryptophan, towards enterocytes’ apical membrane with successive activation of the mTOR pathway, evoking the inflammatory process (Mönkemüller et al., 2020). Notably, its consequences on intestinal permeability and tissue exposition to other microbial toxins, enterocytes’ apoptosis, and microbiome dysbiosis amplifies inflammation from local to systemic coverage (Vodnar et al., 2020). Then, in the presence of diabetes, inflammatory bowel diseases, and obesity as preexistent conditions, microbiome dysbiosis is markedly one of the main elements for enhancing the inflammatory consequences of viral infection.

Recent studies reported polymorphisms in the ACE2 gene in Chinese (Luo et al., 2019), Canadian (Malard et al., 2013), Indian (Patnaik et al., 2014), and Brazilian (Pinheiro et al., 2019) populations. Cao et al. (2020) conducted a study with database analysis and observed that the ACE2 gene has 1,700 variants, of which 11 variants were associated with increased expression of ACE2 in tissues, which can lead to differentiated infectivity by SARS-CoV-2. Different ACE2 variants related to hypertension manifestations were detected and low ACE2 mRNA expression levels were associated with hypertension, dyslipidemia, and heart failure, influencing SARS-CoV-2 infection susceptibility (Cao et al., 2020). In a complementary way, the expression of ACE2 was downregulated in infected cells (Devaux et al., 2020).

Other epidemiological investigations discussed the differential incidence of ACE insertion (I)/deletion (D) polymorphisms among populations and its correlations with viral infection, mortality rate, and recovered clinical cases. In fact, the highest ACE levels were attributed to the dominant allele of ACE I/D polymorphism, revealing a positive correlation between its frequency and infected people in Asian populations. This finding was associated with the presence of comorbidities (hypertension, type-2 diabetes, hyperlipidemia, for example), but no relationships were observed for ACE I/D polymorphisms and the recovery rate of infected patients (Pati et al., 2020). In a complementary way, a clinical study in Spain observed that ACE I/D polymorphisms were related to viral infection, depending on clinical hypertension severity (Gómez et al., 2020), indicating that both genetic and clinical factors are simultaneously implicated in COVID-19 outcomes.

In other analyses, it was reported that ACE2 expression was associated with differences in minor allele frequencies among populations, since Asian people express higher ACE2 levels than Caucasians. Additionally, single nucleotide polymorphisms (SNPs) were investigated for ADAM17 and TMPRSS2, indicating that some TMPRSS2 genotypes are more susceptible to other viral infections, such as H1N1, while ADAM17 expression quantitative trait loci are related to the modulation of the ACE2 shedding process (Brest et al., 2020). This could be explained by the suppression of the ACE2 shedding barrier and the production of soluble form of ACE2 in the presence of a high viral load, which enables infection. Other polymorphisms with effects on COVID-19 progression were also observed in non-coding sequences for cathepsin L, monocyte chemoattractant protein-1 (CCL2), and neutrophil elastase genes, enhancing virus infection and inflammation (Ghafouri-Fard et al., 2020; Vargas-Alarcón et al., 2020).

In addition to expression levels and polymorphisms, ACE2 is extensively regulated by post-translational mechanisms. Those epigenetic modifications include glycosylation, phosphorylation, and shedding processes. In conditions of cellular stress, such as during inflammation, NAD-dependent deacetylase SIRT1 is linked to the ACE2 promoter, favoring its transcription. However, DNA methylation in the ACE2 promoter was lower in lung epithelial cells when compared to other target cells, indicating its high transcription levels in lung tissue, which allows viral infection (Saponaro et al., 2020; Sen et al., 2021).

Taken together, those factors belong to a complex mechanism involving mutations affecting the essential molecular components for viral entry (ACE2, ADAM17, and TMPRSS2); changes in their mRNA expression and post-translational regulation, implicated the severity of clinical symptoms observed in the presence or absence of patients’ comorbidities.

Asselta et al. (2020) compared variations of the TMPRSS2 gene between populations in Italy and other Europeans and Asians. The study indicated that the TMPRSS2 gene and its genetic variants may be promising for disease modulation, suggesting that ACE2 may not be the only target of the virus. In the same way, other genes may be responsible for presenting alterations that may interfere in the individual response to the virus (**Table 1**).

**Table 1:** Location and description of genetic polymorphisms that may be involved with a higher probability of COVID-19 severity.

Locus	Gene	Localization/Function of protein	Polymorphisms + SARS-CoV-2	References
**Xp22.2**	**ACE2**	SARS-CoV-2 receptor, Heart, lungs, nervous system, liver, and kidney cells	**Arg514-Gly** Cardiovascular and pulmonary conditions due to alteration of the AGT-ACE2 pathway	Hou et al. (2020)
			**Ser19-Pro** **Glu329-Gly** Low binding affinity and lack of some of the key residues in the complex formation with the Spike SARS-CoV-2 protein	Hussain et al. (2020)
**2p25.1**	**ADAM17**	Release of ACE2 into the extracellular space Endothelial cell, astrocytes, and neurons	Evaluations of SNPs showed susceptible variations	Li et al. (2014)
**2q24.2**	**DPP4**	SARS-CoV-2 co-receptor Blood circulation and endothelial cells, including capillaries that surround endocrine and intestinal organs	–	Li et al. (2020c)
**15q26.1**	**PCSK3**	Viral pre-activation Pulmonary epithelium and fibroblasts	**Arg298Gln** Influence on the recognition of the target RxxR sequence within the SARS-CoV-2 spike protein	Latini et al. (2020)
**19q13.32**	**ApoE**	Co-expressed where the ACE2 receptor is highly expressed Alveolar type II cells, lung	**e^4^ allele** **Cys112Arg** Serious disease, regardless of pre-existing dementia, cardiovascular disease, and type 2 diabetes	Kuo et al. (2020)
**Xp22.2**	**TLR7**	Essential component of innate immunity intracellularly, in the endosomes	**Val795Phe** Serious disease Allele homozygosis may have an adverse impact on the natural history and prognosis of COVID-19 in males	van der Made et al. (2020)
**4q35.1**	**TLR3**	NF-kappa-B activation, IRF3 nuclear translocation, cytokine secretion, and inflammatory response Endoplasmic reticulum membrane	Ser339fs/WT Pro554Ser/WT Trp769*/WT Met870Val/WT Rare loss-of-function variants	Zhang et al. (2020c)
**11p15.5**	**IRF7**	Key transcriptional regulator of immune responses dependent on interferon type I (IFN) which plays a critical role in the innate immune response against DNA and RNA viruses. It regulates the transcription of IFN type I genes (IFN-alpha and IFN-beta) and genes stimulated by IFN (ISG) by binding to an interferon-stimulated response element (ISRE) in their promoters.	Pro364fs/ Pro364fs Rare loss-of-function variants	
			**Met371Val/** **Asp117Asn** Rare loss-of-function variants	

Human leukocyte antigen (HLA) genes, HLA-A, -B, and -C, among others, are located on chromosome 6 (6p21.34) and encode proteins involved in the processing and presentation of antigens, as well as a wide range of other immunological functions (Parham and Moffett, 2013). The connection of these genes with coronavirus infection was mentioned by Lin et al. (2003), who showed that the smallest binding peptides for SARS-CoV-1 were found for HLA-B * 46: 01. Furthermore, Nguyen et al. (2020) performed an in silico analysis covering the binding affinity of the viral peptide-MHC class I in 145 HLA-A, -B, and -C genotypes and found that HLA-B * 46: 01 also occurred for SARS-CoV-2, suggesting that this allelic variant leads to vulnerability to the disease. However, the HLA B * 15: 03 variant was linked with the ability to present SARS-CoV-2 peptides, suggesting cross-protection with immunity based on T cells. However, Wang et al. (2020c) presented preliminary results, where HLA-A * 11: 01, -B * 51: 01, and -C * 14: 02 alleles showed connection with worse clinical outcomes in patients with COVID-19.

The Severe Covid-19 GWAS Group (2020) performed a meta-analysis of broad genomic association in which they analyzed SNPs in patients with severe acute respiratory syndrome by SARS-CoV-2 and control individuals in Italy and Spain. Two loci associated with respiratory failure in COVID-19 were found, one of which was the GA-G insertion-deletion variant in locus 3p21.31 that is composed of six genes (SLC6A20, LZTFL1, CCR9, FYCO1, CXCR6, and XCR1). Some of these genes are associated with greater severity of COVID-19 symptoms. The SLC6A20 gene synthesizes the SIT1 protein, which together with LZTFL1, interacts with ACE2 cell receptors. The other genes encode chemokine receptors of the C-C and CXC families, which control cell migration associated with immune surveillance by trafficking effector cells to sites of infection and inflammation. According to Zeberg and Pääbo (2020), this genomic sequence, a 49.4 kb haplotype, previously identified by The Severe Covid-19 GWAS Group (2020), was inherited from a Neanderthal who lived in Croatia 50,000 years ago and has a frequency of 30% in the South Asians, 8% in Europeans, and 4% in the American population. Despite the variations in the frequency of this genomic sequence, there are no differences in disease severity around the world due to several non-genetic factors, such as multiple comorbidities, advanced age, physical inactivity, and smoking, among others, which also contribute to respiratory failure.

The other region studied by The Severe Covid-19 GWAS Group (2020) regarding SARS-CoV-2 infection was the SNP A or C at locus 9q34.2, which is related to the ABO blood system, and showed the highest susceptibility of individuals in group A and the lowest susceptibility of individuals in group O. Other studies investigated patients in relation to blood groups and showed that patients in blood group O, regardless of Rh, had a lower incidence of SARS-CoV-2 infection, with individuals in group A being more sensitive and prone to disease, presenting a higher frequency among patients with COVID-19, in addition to being the most serious cases (Göker et al., 2020). The authors suggested that the biological mechanisms included in these results may be related to other biological effects of the identified variant, including stabilization of the von Willebrand factor or the development of neutralizing antibodies against protein-bound N-glycans (The Severe Covid-19 GWAS Group, 2020; Zhao et al., 2020).

Other studies have shown evidence of the increased probability of individuals in blood group A to have COVID-19 (Zietz et al., 2020). A possible explanation for this would be the fact that anti-A antibodies could inhibit the interaction of the Spike protein with the ACE-2 receptor (Guillon et al., 2008). Therefore, blood group O appears to be protective, while group A may be more susceptible to the disease. In this way, patients in group A deserve greater attention when infected by SARS-CoV-2, and further molecular studies are also needed to obtain answers regarding the real protective role of the O antigen (Göker et al., 2020).

Another hypothesis is that susceptibility to SARS-CoV-2 may also be related to the composition of the intestinal microbiota. Based on the proteomic profile of patients with COVID-19, Gou et al. (2020) conducted a study in which they observed the direct relationship between the characteristics of the intestinal microbiota of some healthy individuals, generally older, and the abnormal activation of pro-inflammatory cytokines, which can determine the predisposition to the severe form of COVID-19.

## Perspectives in the Diagnosis and Treatment of COVID-19: the Contribution of Genetic Analysis

Knowledge of the patients’ immune phenotype/genotype is necessary to understand the complexity of COVID-19 (Mangalmurti and Hunter, 2020). Knowing the changes in the signaling pathways caused by the infection can also contribute to the elucidation of the molecular cascades that trigger infection and the severity of the clinical symptoms. So, it is necessary to find molecular targets and alternatives to prevent the progression of COVID-19 (Catanzaro et al., 2020).

Given the information collected since the pandemic began, it is almost certain that the organism’s success in overcoming COVID-19 is linked to the genetics of each individual, their polymorphisms, their patterns of gene expression, and possibly the diversity of intestinal microbiota. However, there are still no parameters to assess genetic markers in individuals. According to Brest et al. (2020), in view of the multifactorial genetic impact for the risk of SARS-CoV-2 infection and disease severity, there is the possibility of assessing SNP profiles of the ACE2, ADAM17, and TMPRSS2 genes in an attempt to identify vulnerable populations, creating a risk score.

A study that is being conducted at the Center for Human Genome and Stem Cell Studies at the University of São Paulo (Brazil) aims to investigate the genome of super-resistant individuals and individuals susceptible to SARS-CoV-2. It is seeking to understand the factors associated with cases of nonagenarians with diabetes and hypertension that have recovered from the disease and young people with no history of chronic diseases that have died. According to the researchers, individuals who develop severe forms may have so-called “risk genes”, while the infected group that does not develop the severe form of the disease, has “protective genes” (Alisson, 2020). An international consortium, the COVID Human Genetic Effort, is currently focused on investigating naturally resistant individuals and healthy young patients who developed severe symptoms. This group of researchers considers these studies a promising tool to understand the genetic determinants of COVID-19 (Casanova et al., 2020).

In this context, the need for genomic analysis and its application in diagnostic medicine is emphasized, since the identification of some genetic patterns can contribute to the clinical evaluation of patients and the amplification of choices for the treatment of each individual. As in other events related to infectious diseases throughout history, this new stage features the possibility to develop new skills and use different tools to guide and assist health professionals in different phases of the treatment and diagnosis of COVID- 19.

## Author Contributions

All authors contributed to the article and approved the submitted version. All the authors contributed equally to this work.

## Funding

The authors are supported by Fundação de Amparo à Pesquisa do Estado de São Paulo (FAPESP) [Grant #2020/05816-2 (MG-C), #2019/20303-4 (DRA) and #2017/13328-5 (RL)], Conselho Nacional de Desenvolvimento Científico e Tecnológico [CNPq proc 307718/2019-0 (DRA)] and Coordenação de Aperfeiçoamento de Pessoal de Nível Superior—Brasil (CAPES)—Finance Code 001 (CTB).

## Conflict of Interest

The authors declare that the research was conducted in the absence of any commercial or financial relationships that could be construed as a potential conflict of interest.
